# Uma Nova Área Promissora

**DOI:** 10.36660/abc.20210764

**Published:** 2021-10-06

**Authors:** Juan Carlos Plana

**Affiliations:** 1 Baylor College of Medicine Texas Heart Institute Houston Estados Unidos da América Baylor College of Medicine, Texas Heart Institute, Houston - Estados Unidos da América

**Keywords:** Ecocardiografia, Insuficiência Cardíaca, Oncologistas

O termo “cardio-oncologia” foi criado há aproximadamente 15 anos. Eu me recordo dos primórdios: última sessão do último dia de encontros científicos locais e internacionais de cardiologia, com a presença principalmente de amigos próximos. Grandes avanços na área de cardio-oncologia ocorreram desde então, com a formação de grupos de interesse e conselhos em nossas sociedades, e desenvolvimento de diretrizes científicas nos guiando na avaliação e no tratamento desses pacientes com condições tão complexas.^1 , 2^ Assim, é com enorme interesse que recebemos o “Posicionamento Brasileiro sobre o Uso da Multimodalidade de Imagens na Cardio-Oncologia - 2021”, um documento abrangente, muito bem escrito, e prático que servirá como um recurso precioso para profissionais de imagem no Brasil e em todo o mundo.

Sinto muito orgulho do que alcançamos até o momento, mas é inevitável pensar no que ainda precisa ser feito nos próximos quinze anos na área de imagem em cardio-oncologia.

A ecocardiografia é e continuará sendo o carro-chefe nesta área. No entanto, ainda existem discordâncias entre oncologistas e cardio-oncologistas sobre o valor de técnicas avançadas como a ecocardiografia 3D e técnica de *strain* . Qual é o problema? Na área de imagem, não estamos acostumados a testar nossas modalidades de imagens quanto ao impacto sobre desfechos duros. Por outro lado, os oncologistas estão acostumados a grandes ensaios clínicos de alta qualidade para testar suas intervenções. Eles esperam que alcancemos os mesmos padrões. Um passo nessa direção foi dado por investigadores do estudo SUCCOUR, em que pacientes foram separados aleatoriamente para realizarem ecocardiografia 3D ou com *strain* para avaliação de insuficiência cardíaca estágio B. Apesar de os autores não terem observado diferenças estatisticamente diferentes no desfecho primário de mudança na ejeção de fração do ventrículo esquerdo (FEVE) entre os dois métodos, um menor número de pacientes preencheram os critérios de disfunção cardíaca relacionada à terapia do câncer durante o acompanhamento no grupo de pacientes com terapia guiada pelo *strain* longitudinal global (SLG). Ainda, a FEVE em um ano foi maior nesse grupo em comparação ao grupo com terapia guiada pela FEVE.^3^

Em relação a outros métodos, vejo um grande futuro para a área de imagens paramétricas na ressonância magnética cardíaca. Quando se detecta disfunção cardíaca, não está claro qual o mecanismo fisiopatológico que levou a essa condição; a presença de edema ou fibrose pode direcionar a duração do tratamento desses pacientes.

A tomografia computadorizada cardíaca continuará sendo muito útil na avaliação de doença cardíaca estrutural, especialmente doença cardíaca induzida por radiação.

Talvez a modalidade de imagem que parece ser menos promissora é a cardiologia nuclear, devido a altas doses de radiação administradas, e o fato de a técnica não oferecer nada que os demais métodos que não utilizam radiação já oferecem.

Possivelmente, a principal pergunta é: que desfechos oncologistas e médicos especialistas em insuficiência cardíaca esperam das técnicas de imagens para incorporá-las nas suas condutas diárias – é mortalidade ou internações por insuficiência cardíaca? Será necessário um acompanhamento em longo prazo de um grande número de pacientes. Eles ficarão satisfeitos com o impacto dessas técnicas sobre o VO2 máximo ou questionários de qualidade de vida? E quanto aos biomarcadores?

Antecipo que os próximos 15 anos serão dedicados à geração de desfechos para as nossas modalidades de imagens. Nós trabalharemos próximos a nossos oncologistas e médicos da insuficiência cardíaca para lhes fornecer o nível de evidência que buscam, levando o campo da Cardio-Oncologia a um novo patamar.
