# Supplementary material for: Uma Nova Área Promissora
Source: Arq Bras Cardiol. 2021 Oct 6;117(4):614. [Article in Portuguese] doi: 10.36660/abc.20210764 (PMC8528356; doi:10.36660/abc.20210764)
Supplement: Translation

# Translation: A Thriving New Field

**Authors:** Juan Carlos Plana

**Affiliations:** 1 United States of America Baylor College of Medicine, Texas Heart Institute, Houston - United States of America

**Keywords:** Echocardiography, Heart Failure, Oncologists

It has been approximately 15 years since the term “cardio-oncology” was coined. I remember the beginnings: last session of the last day in local and international cardiology scientific meetings, with the attendance of mostly close friends. The field of cardio-oncology has advanced considerably since that time, with formation of special interest groups and councils in our societies, and development of scientific statements guiding us as to how to evaluate and treat these very complex patients.^1 , 2^ It is then with great interest that we receive the Brazilian Position Statement on the Use of Multimodality Imaging in Cardio-Oncology -2021, a comprehensive, very well written and practical document that will serve as an invaluable resource to imagers in Brazil and all over the world.

I feel very proud of what we have accomplished but can’t help thinking of what needs to be done in the next fifteen years in the field of Imaging in Cardio-Oncology.

Echocardiography is and will continue to be the working horse in the field. Nevertheless, there are still disagreements between oncologists and cardio-oncologists on the value of advanced techniques like 3D echocardiography and strain Imaging. What is the problem? In the field of imaging, we are not used to putting our imaging modalities to the test looking for an impact on hard endpoints. In contrast, oncologists are used to high-quality large randomized clinical trials to test their interventions. They hold us to the same standards. A step in that direction was taken by the SUCCOUR investigators, randomizing patients to strain vs 3D echocardiography for adjudication of stage B heart failure. Although the primary outcome of change in left ventricular ejection fraction (LVEF) was not significantly different between the two arms, there were fewer patients meeting the cancer therapeutics-related cardiac dysfunction (CTRCD) criteria during follow-up in the global longitudinal strain (GLS)-guided group. Also, the 1-year LVEF was higher in the GLS-guided group when compared with the LVEF-guided arm.^3^

As far as other modalities are concerned, I see a great future for parametric imaging in cardiac magnetic resonance imaging. When cardiac dysfunction is detected, it is unclear what is the pathophysiologic mechanism driving it; the presence of edema or fibrosis may guide treatment duration in these patients.

Cardiac computed tomography will continue to be very helpful in the assessment of structural heart disease especially in the setting of radiation induced heart disease.

Perhaps the imaging modality that appears the least promising is nuclear cardiology, due to the high doses of radiation administered and the fact that they don’t bring to the table anything unique that their non-radiation counterparts can do.

Possibly, the main question is what outcomes oncologists and heart failure doctors expect from imaging modalities to incorporate them in their day-to-day operations – is it mortality or heart failure admissions? A long-term follow-up of a large number of patients will be required. Will they be satisfied with the impact of these techniques on VO_2_max or quality of life questionnaires? What about biomarkers?

I anticipate that the next 15 years will be devoted to the generation of outcome data for our modalities. We will work closely with our oncologists and heart failure doctors to provide them with the level of evidence that they are looking for, taking the filed of Cardio-Oncology to the next level.
